# The Challenge of Illusory Perception of Animals: The Impact of Methodological Variability in Cross-Species Investigation

**DOI:** 10.3390/ani11061618

**Published:** 2021-05-30

**Authors:** Maria Santacà, Christian Agrillo, Maria Elena Miletto Petrazzini

**Affiliations:** 1 Department of General Psychology, University of Padova, 35131 Padova, Italy; christian.agrillo@unipd.it; 2 Padua Neuroscience Center, University of Padova, 35131 Padova, Italy; 3 Department of Biomedical Sciences, University of Padova, 35131 Padova, Italy; mariaelena.milettopetrazzini@gmail.com

**Keywords:** visual illusions, comparative perception, motion illusions, distortion illusions, subjective contours

## Abstract

**Simple Summary:**

Research in neurobiology and ethology has given us a glimpse into the different perceptual worlds of animals. More recently, visual illusions have been used in behavioural research to compare the perception between different animal species. The studies conducted so far have provided contradictory results, raising the possibility that different methodological approaches might influence illusory perception. Here, we review the literature on this topic, considering both field and laboratory studies. In addition, we compare the two approaches used in laboratories, namely spontaneous choice tests and training procedures, highlighting both their relevance and their potential weaknesses. Adopting both procedures has the potential to combine their advantages. Although this twofold approach has seldomly been adopted, we expect it will become more widely used in the near future in order to shed light on the heterogeneous pattern observed in the literature of visual illusions.

**Abstract:**

Although we live on the same planet, there are countless different ways of seeing the surroundings that reflect the different individual experiences and selective pressures. In recent decades, visual illusions have been used in behavioural research to compare the perception between different vertebrate species. The studies conducted so far have provided contradictory results, suggesting that the underlying perceptual mechanisms may differ across species. Besides the differentiation of the perceptual mechanisms, another explanation could be taken into account. Indeed, the different studies often used different methodologies that could have potentially introduced confounding factors. In fact, the possibility exists that the illusory perception is influenced by the different methodologies and the test design. Almost every study of this research field has been conducted in laboratories adopting two different methodological approaches: a spontaneous choice test or a training procedure. In the spontaneous choice test, a subject is presented with biologically relevant stimuli in an illusory context, whereas, in the training procedure, a subject has to undergo an extensive training during which neutral stimuli are associated with a biologically relevant reward. Here, we review the literature on this topic, highlighting both the relevance and the potential weaknesses of the different methodological approaches.

## 1. Introduction

Understanding how the different animal species see the world around them has long interested researchers from ancient times to today. For many animals, vision is the primary link to the world that allows them to seek out food, communicate, avoid predators, or find a mate to reproduce. Since their appearance, animals have colonized nearly every ecological niche on Earth and, thus, have evolved different visual systems to assimilate the surrounding information. For example, some species can only see in shades of grey, whereas other species have colour vision even in a near-total darkness condition. Other species can see different parts of the light spectrum that are invisible to humans, such as ultraviolet and infrared light [1]. As a consequence, the animal visual worlds are highly different from the world that humans take for granted. However, perceiving the world is not a simple and passive acquisition of the images of the surrounding environment, but it is based on past experience and stored information [2]. In fact, a three-dimensional image has to be translated into a bi-dimensional retinal representation that, subsequently, is interpreted by cognitive and neural processes. The perception of a visual stimulus can, therefore, differ considerably from its physical counterpart. In the psychology literature, some of these “errors” of perception are referred to as visual illusions. Contrary to this indirect approach, it has been proposed that the nature of visual perception is not limited to simple geometric or physical properties. In fact, according to the Gibson’s ecological approach, perception is a direct contact with the environment and, thus, not mediated by mental images or other mental representations [3,4,5]. Gibson also stated that the content of perception must be already relevant for action, and it is primarily comprised of opportunities for behaviour. According to this direct approach, vision must be considered in terms of the whole visual system and activity over time, and it is insufficient to look at its retinal image to understand what an animal perceives.

**Figure 1:**
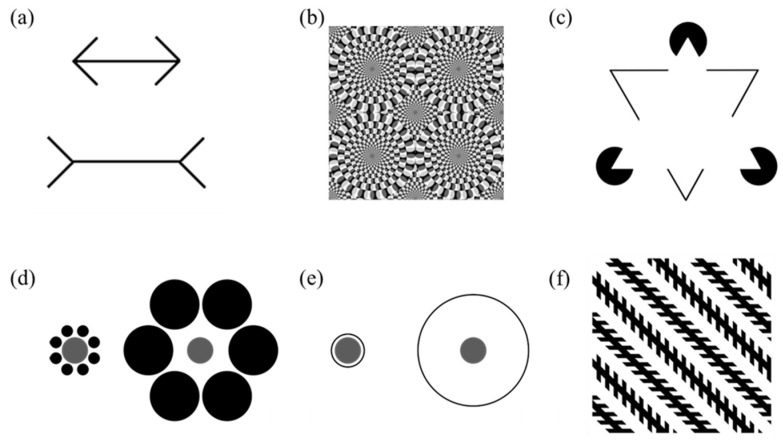
Müller-Lyer illusion (**a**), Rotating Snake illusion (**b**), Kanizsa’s triangle (**c**), Ebbinghaus illusion (**d**), Delboeuf illusion (**e**) and Zöllner illusion (**f**).

The research regarding the perception of visual illusions in animals began in the 1920s [6], and since then, visual illusions have become an important tool to compare visual perception in animals. This research, in fact, allows researchers to assess whether animals interpret and alter visual inputs as humans do or if they detect visual inputs with little or no variability. In addition, visual illusions can be also used to comprehend the psychological and cognitive processes underlying visual perception and to shed light on the impact of environmental and evolutionary pressures on visual perception and processing [7]. In fact, according to the traditional theories of perception, the evolutionary assumption underlying these studies is that, if the susceptibility to the same illusory pattern is shared between two species, it is possible to infer that the two species share similar perceptual mechanisms to perceive the world around them. Interspecific comparisons have led to a high heterogeneity in the results. Some studies reported a similar perception to the same illusory pattern in distantly related species. For instance, different monkeys (capuchin monkeys, *Cebus apella* [8]; rhesus monkeys, *Macaca mulatta* [9]), birds (ringneck dove, *Streptopelia risorii* [10]; African grey parrot, *Psittacus erithacus* [11]) and fish (guppies, *Poecilia reticulata* [12]; redtail splitfin fish, *Xenotoca eiseni* [13]) demonstrated an ability to perceive the Müller-Lyer illusion as humans do. Figure 1a shows the classical version of this illusion in which two parallel lines are presented; one line has inward-pointing arrows on the ends, whereas the other has outward-pointing arrows. Humans and the abovementioned species were shown to underestimate the length of the target line ending with the outward-pointing arrows and to overestimate the length of the target line ending with the inward-pointing arrows. In addition, rhesus monkeys [14], felines (lions, *Panthera leo* [15]; cats, *Felis catus* [16]) and fish (guppies and zebrafish, *Danio rerio* [17]) seem to be susceptible to motion illusions, perceiving the Rotating Snake illusion (Figure 1b). Lastly, chimpanzees (*Pan troglodytes* [18]) and other monkeys (for a review, see [19]), cats [20], birds (barn owl, *Tyto alba* [21]), fish (bamboo sharks, *Chiloscyllium griseum* [22]; goldfish, *Carassius auratus* [23]; redtail splitfin [24]) were shown to perceive illusory contours of the Kanizsa illusory picture (Figure 1c).

Other studies, however, highlighted a lack of perception of an illusory pattern in different vertebrates. Guinea baboons (*Papio papio* [25]) and starlings (*Sturnus vulgaris* [26]) do not see the Ebbinghaus illusion, whereas dogs (*Canis lupus familiaris* [27,28]) and red-footed tortoises (*Chelonoidis carbonaria* [29]) do not see the Delboeuf illusion. Both illusions occur when the size of a target item is misperceived depending on its surrounding context. Figure 1d,e show the classical versions of both illusions: two identical target circles encompassed by a larger and smaller context, namely rings in the Delboeuf illusion and circles in the Ebbinghaus illusion. In both cases, humans typically underestimate the size of the target circle encompassed by the larger context and tend to overestimate the size of the target circle encompassed by the smaller context. In addition, dogs [30] and bamboo sharks [22] do not perceive the Müller-Lyer illusion. 

Lastly, other studies reported the perception of visual illusions in the opposite direction compared to humans, also defined as a reversed perception. Pigeons (*Columba livia* [31]) and bamboo sharks [32] experienced a reverse Ebbinghaus illusion, whereas different species of teleost fish perceive a reverse Delboeuf illusion [33,34]. In addition, both pigeons [35] and bantam chickens (*Gallus gallus* [36]) demonstrated a reverse Zöllner illusion that consists in a set of parallel lines that appear non-parallel due to series of short crosshatches superimposed on the lines (Figure 1f).

In summary, some studies found a similar perception of the same visual illusion among vertebrates, suggesting similar perceptual mechanisms, whereas others highlighted interspecific differences. The origin of the observed variability in the perception of visual illusions remains unclear. The most accredited hypothesis is that this observed variability might be ascribed to different contextual factors, such as the adopted methodology, the different stimuli, the age and the sex of the subjects (e.g., [7,37]). In fact, there is evidence that different methods of investigating the Ebbinghaus illusion can lead to different results in the same species, as in bantam chickens. Rosa Salva and colleagues [38] demonstrated that four-days chicks perceived the illusion as humans do, whereas Nakamura and colleagues [39] concluded the opposite testing 6-month-old chickens. In addition, in some cases (e.g., the odd task used with olive baboons or *Papio anubis* in the case of the Zöllner illusion [40]), the methodology adopted provides a yes/no response in terms of susceptibility to an illusory phenomenon, but the information about the direction of the illusion (human-like or reverse) is not available.

In the literature on illusory perception, almost every study, with a couple of exceptions, has been conducted in laboratories adopting two different methodological approaches, namely spontaneous choices tests and training procedures. Due to the primary relevance of the abovementioned methodological questions, here, we critically examine and compare behavioural studies that investigated the perception of visual illusions in non-human animals. In this review, after a brief description and presentation of various studies using the different methodologies, we compare these different methods, highlighting the pros and cons of each.

## 2. Field Studies

When breeding, feeding and moving within their environment, wild animals interact with their physical surroundings and the biological world. In almost every field study, researchers simply observed animals in their natural environment without any manipulation. In few cases, researchers could adopt a minimally invasive approach deciding to move or manipulate some physical characteristics of the environment to observe any consequent change in an animal’s behaviour. Field studies have been conducted also to understand whether animals perceive or use visual illusions. In fact, visual illusions are naturally present in real-life contexts and might be advantageous and positively selected in animal species able to generate them by manipulating the environment or their coloration and movement.. For example, illusions of size or brightness may be present on the body and influence different social context such as choosing a mate or intrasex conflicts. In fact, in both sexual and conflict contexts, animals typically compare locally available conspecifics and make choices based on size and colour that may signal quality [41]. Such illusions may change the perceived quality of an individual and, thus, influence animals’ behaviour. Instead, motion illusions or illusions that obscure the body shape might play an important part in animal camouflage to reduce the risk of being predated or to increase the possibility to predate [42]. Despite their high ecological value, there is only a little and mainly indirect evidence of illusory phenomena in real-life contexts with only one exception. 

One indirect evidence regards male fiddler crabs (*Uca mjoebergi*) that actively manipulate their social environment, namely the presence of neighbours, that resembles the Ebbinghaus illusion, to potentially increase their relative attractiveness [43]. In addition, male guppies may actively utilize the same illusory pattern to select the most appropriate context relative to their coloration to increase their reproductive success [44]. The only direct evidence is the study in the great bowerbird (*Ptilinorhynchus nuchalis*), in which the authors demonstrated that males construct bower courts with forced visual perspective from the audience view to manipulate their size perception and, thus, to increase their reproductive success [45]. To create this perspective illusion, the males of this species arrange grey and white objects in a positive size–distance gradient forced perspective. The authors demonstrated that, when the forced perspective is reversed, males actively restore it within a couple of days [45]. In addition, males vary consistently in the quality of the perspective illusion and that the latter is positively correlated with mating success [46,47].

## 3. Laboratory Studies

The environmental conditions cannot be neatly controlled and documented; in fact, the field studies are characterized by a lack of control and the difficulty of precisely distinguishing the several environmental factors. This is the main reason why the majority of the studies regarding the perception of visual illusions have been conducted in laboratories. In this controlled environment, researchers adopted two different methodological approaches that differ in several respects which we will later discuss.

### 3.1. Spontaneous Choice Tests

In spontaneous choice tests, subjects are typically presented with biologically relevant stimuli that, in most cases, consist of food items (Table 1). However, the experimental setup considerably differs depending upon the type of visual illusion. 

**Figure 2:**
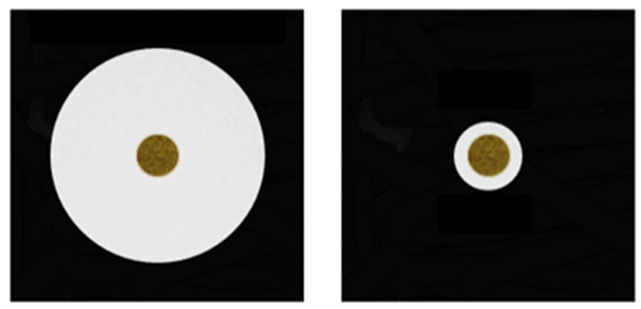
An example of biologically relevant stimuli (i.e., food) that could be used in spontaneous choice studies investigating the Delboeuf illusion in animals.

Regarding all the illusions in which the visual context induces a distortion in size, the procedure consists in the presentation of two arrays containing two identical food portions in two different contexts that resemble a specific illusory pattern. The first food portion selected by the subject is recorded as the dependent variable. This approach exploited animals’ natural tendency to maximise the food intake. Different studies proved the spontaneous preference for larger food portions in several animal species such as chimpanzees [54], cats [55], African grey parrots [56], Italian wall lizards (*Podarcis sicula* [57]), Hermann’s tortoises (*Testudo hermanni* [58]) and guppies [59]. Moreover, this approach relies on the evidence that humans’ perception of food size is influenced by the context in which it is presented, in particular, in a Delboeuf illusory context (Figure 2). 

Different studies demonstrated that humans overestimate a food portion size and, hence, under-serve when it is presented on a smaller dish (e.g., [60,61,62]). The pioneering study adopting this procedure in non-human animals was conducted with chimpanzees by Parrish and Beran [48] to assess the existence of the Delboeuf illusion in this species. When presented with two identical food portions on two different-sized plates, chimpanzees significantly selected the food portion inserted in the smaller plate, thus perceiving the Delboeuf illusion as humans do. Chimpanzees were also presented with control trials consisting of two different-sized food portions on two identical plates to assess their tendency to maximise the food intake in the experimental context. The assumption of this approach is that, if a species spontaneously selects the larger quantity in order to maximize food intake, it is expected to choose the portion that appears larger in illusory trials. Other studies adopted the same methodological approach of Parrish and Beran [48] to investigate the perception of the Delboeuf illusion in cats [50], dogs [28], reptiles [29,51] and fish [33,34]. The same approach has been used to study other visual illusions, such as the Müller-Lyer illusion in reptiles [52] and horses (*Equus caballus* [53]). A crucial aspect of this methodological approach consists in the performances in control trials in which two different-sized food portions are presented. As abovementioned, this type of procedure exploits animals’ natural tendency to maximise the food intake. Nonetheless, animals could also not choose the larger of two rewards. This could occur when both food portions are large enough for the species under investigation and, thus, animals do not need to maximize their intake (e.g., [28,49]). Alternatively, animals could be trying to maximize food intake, but the physical difference between the food portions could be too subtle to be detected due to their visual acuity (e.g., [28,49]). In this scenario, since animals are not maximising the food intake in the experimental context, no conclusion can be drawn regarding their performances in illusory trials. 

There is also a simpler procedure to investigate the perception of visual illusions adopting a spontaneous approach; however, this can be applied only to motion illusions, such as the Rotating Snake illusion (Figure 1b). The assumption is that a subject perceives the motion illusion, and it is expected to approach, more than chance, the illusory stimulus to pursue movement. In the only two studies of this type, the researchers investigated the perception of the Rotating Snake illusion in felines, namely cats [16] and lions [15], adopting a preferential looking experiment. In both studies, researchers placed different visual stimuli in their environment, respectively, a “cat-café” in Fukuoka-city and an Italian zoological garden. The stimuli consisted of the Rotating Snake illusion and a control stimulus that did not evoke any motion perception, even if the overall configuration was identical to that of the illusory pattern. Researchers recorded the interacting time or the number of interactions with the illusory stimuli and found that both cats and lions were specifically attracted by the Rotating Snake illusion to pursue movement. In addition, Regaiolli and colleagues [15] also found that this illusion has a positive effect on the animals’ welfare, consisting of a reduction in self-directed behaviours and an increase in attentive behaviours. 

However, confounding factors can potentially affect spontaneous choice tests. In fact, animals may have a bias for choosing the illusory stimulus more than chance, even if they do not perceive the illusion. This emerged in a study investigating the perception of the Delboeuf illusion in ring-tailed lemurs (*Lemur catta* [49]). As in the previous studies, lemurs were presented with both control and illusory trials. Despite they exhibited poor performance in control trials compared to other mammals previously observed, one subject consistently selected the food portion in the larger plate in the illusory trials. This might lead researchers to believe that this lemur was highly sensitive to trials in which the plates were differing in size. In fact, this condition was less frequent compared to control trials, and it may have led to a bias for choosing one array (in this case, the food portion on the larger plate), which was not observed when identical plates were presented. The same hypothesis of a spontaneous bias for the surrounding context, itself, also emerged in the study with guppies that demonstrated to strongly perceive a reverse Delboeuf illusion [33]. However, in this investigation, the researchers conducted a control experiment in which the test trials consisted in presenting the large array and the small array without any food stimuli. If guppies exhibited a spontaneous preference for the larger array in the presence of different-sized arrays, they were expected to select this one more than chance. The results of this control experiment excluded the possibility that the guppies’ performance in illusory trials was due to any sort of spontaneous bias for the context in which the food portion were presented [33]. Another confounding factor that can potentially affect a spontaneous choice test consists in the foraging habits of a species. For example, in the reptiles’ studies, tortoises exhibited a lower performance compared to bearded dragons in size estimation with vertically arranged items [63]. An intriguing possibility is that tortoises pay less attention to vertical stimuli in the natural environment due to their ecological niche, since they live on the ground layer. Bearded dragons, instead, are considered to be semi-arboreal, and they quite readily climb and bask at height. Thus, the configuration or the position of the stimuli could influence the test result [37,63]. 

In summary, studies using the spontaneous procedure reported the perception of visual illusions in a wide range of species. However, as the natural behaviour of the animals is observed, it exists the possibility that animals’ choices are not based on the target stimulus, i.e., food portion, but on the context in which the stimuli are presented, potentially resulting in a “false” illusory perception. For better control this confounding factors, other studies preferred to use training procedures with inanimate objects as stimuli.

### 3.2. Training Procedures

In a training procedure, a subject undergoes an extensive training during which neutral stimuli are associated with a biologically relevant reward. In fact, a subject is trained to select a target stimulus in order to obtain a reward (i.e., food or social companions). This procedure necessarily requires several training phases before the subject is presented with a visual illusion. In the first phase, a subject usually familiarizes with the apparatus and the procedure undergoing a shaping procedure in order to learn the association of the reward with the correct response. Subsequently, the subject is presented with a discrimination task until it reaches a learning criterion—normally a significant percentage of correct choices in two consecutive sessions or an overall significant performance considering all training trials (e.g., [12,27]). 

In this type of procedure, the stimuli can be presented on a monitor, as it has been successfully done with several species such as primate and non-primate mammals (e.g., [27,30,64,65]), birds (e.g., [35,36]) and fish (e.g., [17,22,32]) (Table 2). Alternatively, the stimuli can be printed on specific presentation cards; this setup has mainly been adopted with fish (e.g., [12,13,24]) but also with birds (e.g., [38]).

The majority of the studies adopted a training procedure consisting of a two-choice discrimination task (Table 2). In particular, a subject is simultaneously presented with two stimuli that differ in regard to their size, brightness or other physical characteristics. Parrish and colleagues [65] trained rhesus monkeys and capuchin monkeys to investigate whether they perceive the Delboeuf illusion. In the first experiment, monkeys were presented with a relative discrimination task requiring them to choose the larger of two target dots that were sometimes encircled by concentric rings. All monkeys successfully learned the discrimination rule; instead, in illusory trials, they exhibited heterogenous results. In fact, two monkeys demonstrated to perceive the illusion as humans do; twelve monkeys seemed not to perceive the illusion, whereas six appeared to perceive the illusion in a reversed manner [65]. As the abovementioned spontaneous choice examples, the researchers were concerned that the contrasting results were driven by a bias for the dot encircled by a large context considering the contexts and dots as one stimulus. For this reason, they conducted a second experiment with an absolute classification task in which subjects had to classify a target dot of variable size as either “large” or “small” in comparison with a never-presented central target size. In this task, capuchin and rhesus monkeys showed sensitivity to the Delboeuf illusion as reported for humans, demonstrating that the perception of this illusion is influenced by the procedure adopted. Despite the absolute classification procedure proved to overcome the aforementioned concerns, it has been less adopted than the relative two-choice discrimination procedure. To date, it had been mainly used with birds, namely pigeons and bantams (e.g., [31,39,66]). Regarding this issue, a recent study from Qadri and Cook [26] with starlings suggests that the choice for the array that is supposed to reflect an illusory perception could be a consequence of the training procedure adopted. In fact, the researchers discovered that the classical training procedure used to investigate this illusion in pigeons leads the starlings to integrate the irrelevant context into their decision process, precluding the study of illusory perception [26]. Thus, the absolute discrimination task also cannot completely resolve or eliminate the abovementioned concerns.

Training procedures have been used to study the perception of visual illusions in non-human animals with a food reward or a social reward (Table 2). The food reward has been used with several different species, from primates to fish. Just to give some examples, Fagot and Tomonaga [18] trained chimpanzees, delivering a piece of apple after a correct choice, whereas Fuss and colleagues [22] trained sharks, rewarding them with dropped food, namely a bait, for a correct choice during the training phase. To date, a social reward was only used in the studies investigating the perception of the Kanizsa illusory picture, the Ebbinghaus illusion and the Müller-Lyer illusion in redtail splitfin fish [13,24,67]. In these experiments, fish were removed from their social group and placed in an unfamiliar square environment. They could rejoin companions only by passing through one of two identical tunnels, that were associated with geometric figures, at opposite corners. Only the door associated with the reinforced stimulus allowed the subject to rejoin its companions.

## 4. A Comparison of the Different Methodological Approaches 

Field research is research conducted in a natural environment or in the real world or simply observing, interpreting and explaining what already exists or manipulating some physical characteristics of the environment to observe any consequent change in an animal’s behaviour. Field study subjects may not be aware that they are being tested. Thus, field studies have the advantage of representing real-life circumstances, because they consist of a range of situations and environments that are encountered in the natural habitat, such as in the studies with the great bowerbird [45,46,47]. For these reasons, this type of study has the highest ecological validity. Laboratory research, on the other hand, is performed in an environment specifically designed for research. This type of research is sometimes characterized as a tightly regulated investigation in which the researcher manipulates the factor under investigation to see whether the manipulation causes a shift in the subjects. Laboratory studies have the advantage of providing better control over irrelevant variables that may otherwise influence the findings, as well as clearer clues to the observed behaviour (e.g., [12,30]). However, laboratory research, like any other form of research, may have drawbacks. On one hand, the experimental setup can be meticulously monitored and recorded, but, on the other, it could reflect a simulated environment that influences how subjects act and, as a result, alters their performance. Nonetheless, since researchers can usually exert a greater control in a laboratory setting than in a naturalistic setting, the perception of visual illusions in non-human animals has been mainly investigated in laboratories.

Laboratory studies adopt two different methodological approaches: a spontaneous choice test or a training procedure. The two approaches are different in many aspects and also reveal different aspects of visual processing [68]. Animals are expected to behave naturally in spontaneous choice experiments, and their performance is thought to reflect the cognitive and perceptual processes they would activate in nature. However, this procedure has some weakness. In fact, since the stimulus to discriminate corresponds with the reward, the performance of the subjects in this approach is heavily influenced by their motivation. For example, in a food discrimination task, animals may get a certain amount of food regardless of the correctness of their choices; thus, their motivation can decrease trial after trial, leading to a null result, as in the investigation of the Delboeuf illusion in lemurs [49]. In training procedures, on the other hand, animals can only obtain a food reward if they make the right choice. Since only those subjects that successfully learn the discrimination rule (e.g., choose the larger stimulus) are presented with illusory trials, their performances in this type of trials are thought to reflect their real illusory perception. In spontaneous choice tests, animals can also differ in their individual preferences for the stimuli’s distinctive features. This has been found both in interspecific and intraspecific studies. For example, despite the willingness to adopt the same methodological approach, tortoises and bearded dragons had to be tested with different preferred food, respectively, mango jelly and vegetable extract (kale, cucumber and mint) jelly, when tested for the perception of the Delboeuf illusion [29,52]. In the investigation of the same illusion in cats, prior to the experiment, the authors presented two different types of food stimuli, namely canned tuna and dry cat food, to assess any individual difference in the food preference [50]. This allowed the researchers to test each cat with its preferred food [50]. Other sensory modalities, such as olfactory cues, may also affect the animals’ performance in spontaneous choice experiments. In fact, there are high interspecific differences in the relative importance of vision and other senses, such as olfaction, in solving different cognitive tasks. In the field of illusory perception, this could have a crucial impact on the performance in illusory trials. As a matter of fact, the illusions in which the visual context induces a distortion in size are resembled by presenting the same food portion but in two different contexts, as reported above. In such illusory trials, animals that mainly rely on olfaction to select the larger food portion may not be fooled by the visual illusion, leading to a null result [28]. On the contrary, in training procedures, the problem of individual preferences or using other sensory modalities is generally not a concern, since the stimuli are bi-dimensional (i.e., static objects presented on a monitor or printed stimuli). Finally, since the same neutral stimuli may be used with distantly related species, training procedures allow a fine interspecific comparison, as in the studies with the Rotating snake illusion [14,15,16,17]. In contrast, the most appropriate stimuli for the species under investigation are needed in spontaneous choice experiments, as mentioned above. More practical considerations, such as sample size, distinguish the two methods. A larger sample size is normally evaluated in spontaneous choice experiments to evaluate the group performance and, thus, overcome any individual preference for the stimuli. Finally, because motivation may decrease during the trials, a between-subjects design with each subject tested in a relatively short period of time should be preferred in spontaneous choice tests (e.g., [28,33,48]). In training procedures, on the other hand, each subject may require a significant amount of time to meet the learning criterion; thus, for this second methodological approach, a within-subjects design is preferred, as in the dogs study regarding their perception of the Delboeuf and Ebbinghaus illusion [27]. 

The two approaches are clearly complementary. Only a few studies adopted both procedures to have an integrated result regarding the illusory perception of a visual illusions. This is the case of the Delboeuf illusion in guppies [33] and in dogs [27,28]. In a first experiment, Lucon-Xiccato and colleagues [33] trained guppies in a two-choice task to discriminate between two different-sized printed stimuli. Upon achieving the learning criterion, guppies passed to the test phase in which they could face different types of trials, such as congruent (i.e., a large context included the larger target circle and a small context included the smaller target circle) and incongruent (i.e., a large context that included the smaller target circle and a small context that included the large target circle) size discrimination trials. If guppies were susceptible to the Delboeuf illusion, they were expected to be facilitated in size judgments in incongruent trials compared to the congruent trials. The guppies’ performances in these trials revealed that guppies showed a facilitation effect due to the Delboeuf illusion in the reversed direction compared to humans [33]. However, in the illusory trials of the test phase, the authors found no significant preference, both in group analysis considering the entire sample of subjects and in a tentative analysis at the individual level. To further investigate and understand their perception of the Delboeuf illusion, the researchers conducted a second experiment, adopting a spontaneous food choice test in which guppies demonstrated a preference for the larger food item in control trials with a higher accuracy compared to the first experiment (66% vs. 57% [33]). In the illusory trials of this second experiment, guppies showed a marked preference for the food item presented in the large context, revealing a Delboeuf illusory effect greater than observed in the training experiment. This could be related to the higher salience of the stimuli in the spontaneous choice experiment; in addition, guppies could use different strategies to distinguish between printed stimuli and food items, and the difference in illusory effect could be due to distinct strategies [33]. Considering the dogs studies, Miletto Petrazzini and colleagues [28] found that, when dogs could choose between two identical food portions but presented on a Delboeuf illusion context in a spontaneous preference paradigm, they did not seem to be susceptible to the Delboeuf illusion. In line with this conclusion, Byosiere and colleagues [27] used an operant conditioning procedure to train dogs to make a fine discrimination between a larger and a smaller black target circle. The dogs in this experiment also performed randomly at the group level in the presence of the Delboeuf pattern. In both cases, it is intriguing and suggestive that two experiments that used two different approaches (untrained behaviour with biologically relevant stimuli vs. trained behaviour with two-dimensional figures on the screen) came up with the same result. To date, no other illusory perception has been investigated with both procedures, leaving uncertain the obtained results.

## 5. Conclusions

The findings obtained until now prompted a debate as to whether animal species share similar perceptual mechanisms underlying visual perception and if these are homologous to our perceptual mechanisms. This issue becomes even more relevant regarding the possibility of developing animal models to investigate visual perception disorders, to find out general principles necessary to build successful artificial visual systems and, more generally, to study critical research topics in perception that, for practical and ethical reasons, cannot be easily deepened in our species, such as the ontogeny of perceptual mechanisms and the exact neural circuits underlying visual perception. The origins of the heterogeneous perception of visual illusion could be ascribed to the different contextual factors. In this review, it was demonstrated that different methods can lead to different results in the same species. We also identified different aspects of the same methodological approach that should be carefully taken into account when comparing the results of different studies. Lastly, we highlighted both the relevance and the potential weaknesses of the different methodological approaches. Regarding the laboratory studies, we suggest that adopting the two different procedures, namely spontaneous choice tests or training procedures, has the potential to combine their advantages we have discussed in this review. On this basis, despite the fact that spontaneous choice tests or training procedures have been poorly adopted in the same investigation, we expect this twofold approach will become more widely used in the near future in order to shed light on the heterogeneous pattern observed in the literature of visual illusions.

## Figures and Tables

**Table 1:** Summary of the existing works on visual illusions adopting a spontaneous choice paradigm.

Visual Illusion	Reference	Sample	Stimuli	Susceptibility
Delboeuf illusion	[48]	3 *Pan troglodytes* (chimpanzees)	Food	Yes
	[49]	9 *Lemur catta* (ring-tailed lemurs)	Food	n/a
	[50]	18 *Felis catus* (cats)	Food	Yes
	[28]	13 *Canis lupus familiarias* (dogs)	Food	No
	[51]	12 *Pogona vitticeps* (bearded dragons)	Food	Yes
	[29]	8 *Chelonoidis carbonaria* (red-footed tortoises)	Food	No
	[33]	12 *Poecilia reticulata* (guppies)	Food	Reversed
	[34]	12 *Betta splendens* (Siamese fighting fish), 12 *Danio rerio* (zebrafish), 12 *Pterophyllum scalare* (angelfish), 12 *Trichopodus trichopterus* (three-spot gourami) 12 *Xenotoca eiseni* (redtail splitfin)	Food	Reversed
Müller-Lyer illusion	[52]	12 *Pogona vitticeps* (bearded dragons)	Food	Yes
	[53]	9 *Equus caballus* (horses)	Food	Yes
Rotating Snake illusion	[16]	11 *Felis catus* (cats)	Printed stimuli	Yes
	[15]	3 *Panthera leo* (lions)	Printed stimuli	Yes

**Table 2:** Summary of the existing works on visual illusions adopting a training procedure.

Visual Illusion	Reference	Sample	Stimuli	Task type	Reward	Susceptibility
Delboeuf illusion	[65]	7 *Macaca mulatta* (rhesus monkeys)	Presented on a monitor	Two-choice discrimination task/ absolute classification task	Food	No (two-choice task). Yes (absolute classification task)
	[27]	8 *Canis lupus familiaris* (dogs)	Presented on a monitor	Two-choice discrimination task	Food	No
Ebbinghaus illusion	[27]	8 *Canis lupus familiaris* (dogs)	Presented on a monitor	Two-choice discrimination task	Food	Reverse
	[39]	3 *Gallus gallus* (bantams)	Presented on a monitor	Absolute classification task	Food	Reverse
	[31]	6 *Columba livia* (pigeons)	Presented on a monitor	Absolute classification task	Food	Reverse
	[38]	24 *Gallus gallus* (chicks)	Printed stimuli	Two-choice discrimination task	Food	Yes
	[26]	5 *Sturnus vulgaris* (starlings)	Presented on a monitor	Absolute classification task	Food	No
	[32]	4 *Chiloscyllium griseum* (bamboo sharks), 5 *Chromis chromis* (damselfish)	Presented on a monitor	Two-choice discrimination task	Food	Reverse (sharks). Yes (damselfish)
	[13]	8 *Xenotoca eiseni* (redtail splitfin)	Printed stimuli	Two-choice discrimination task	Social	Yes
Müller-Lyer illusion	[30]	7 *Canis lupus familiaris* (dogs)	Presented on a monitor	Two-choice discrimination task	Food	No
	[66]	4 *Columba livia* (pigeons)	Presented on a monitor	Absolute classification task	Food	Yes
	[67]	6 *Xenotoca eiseni* (redtail splitfin)	Printed stimuli	Two-choice discrimination task	Social	Yes
	[12]	12 *Poecilia reticulata* (guppies)	Printed stimuli	Two-choice discrimination task	Food	Yes
	[22]	9 *Chiloscyllium griseum* (bamboo sharks)	Presented on a monitor	Two-choice discrimination task	Food	No
Kanizsa figures	[18]	2 *Pan troglodytes* (chimpanzees)	Presented on a monitor	Two-choice discrimination task	Food	Yes
	[24]	7 *Xenotoca eiseni* (redtail splitfin)	Printed stimuli	Two-choice discrimination task	Social	Yes
	[22]	9 *Chiloscyllium griseum* (bamboo sharks)	Presented on a monitor	Two-choice discrimination task	Food	Yes
Zöllner illusion	[64]	6 *Macaca mulatta* (rhesus monkeys)	Presented on a monitor	Two-choice discrimination task	Food	Yes
	[40]	2 *Papio anubis* (olive baboons)	Printed stimuli	Oddity task	Food	Yes
	[35]	6 *Columba livia* (homing pigeons)	Presented on a monitor	Two-choice discrimination task	Food	Reversed
	[36]	3 *Gallus gallus* (bantam chickens)	Presented on a monitor	Two-choice discrimination task	Food	Reversed
Rotating Snake illusion	[17]	12 *Danio rerio* (zebrafish), 12 *Poecilia reticulata* (guppies)	Presented on a monitor	Two-choice discrimination task	Food	Yes

## Data Availability

Not applicable.
